# Tin fractionation analysis in sediment samples via on-line ID ETV/ICP-MS

**DOI:** 10.1007/s00216-025-06064-y

**Published:** 2025-08-21

**Authors:** Vera M. Scharek, Jens Pfeifer, Jochen Vogl, Heike Traub, Björn Meermann

**Affiliations:** Richard-Willstätter-Straße 11, 12489 Berlin, Germany

**Keywords:** Tin, Organotin compounds, Sediment, Fractionation, On-line IDMS, ETV/ICP-MS

## Abstract

We report a quantification approach for directly determining total tin and tin-based pollutants/species in sediments via electrothermal vaporization/inductively coupled plasma-mass spectrometry (ETV/ICP-MS) utilizing an on-line isotope dilution mass spectrometry (IDMS) approach. The method was developed and validated using an estuarine sediment reference material (BCR-277R), yielding a recovery of 106%. A relative standard deviation (RSD) of 14%, comparable to published data using a similar method, was obtained. A limit of quantification (LOQ) was estimated at 0.008 mg Sn kg^−1^ and sufficient for quantifying the total tin mass fraction of surface sediments along the tidal River Elbe course. Hereby, a decrease towards the river mouth, presumably due to dilution effects by less polluted marine sediment, was observed. Besides total tin, monitoring of organotin compounds (OTCs)/species is of interest in sediments due to their toxic effects on aquatic life. The method’s capability was extended by separating an OTC fraction in a sediment certified reference material (CRM) through the ETV temperature program. While spiking experiments with OTC standards confirmed the assignment, only a small fraction of the total certified OTC amount (3%), likely due to matrix effects, was recovered. However, applying the method to real-world samples, OTCs were detectable along the River Elbe course. By this, we demonstrated the potential of our method as a complementary fast-screening approach to species-specific analysis procedures.

**Graphical Abstract:**

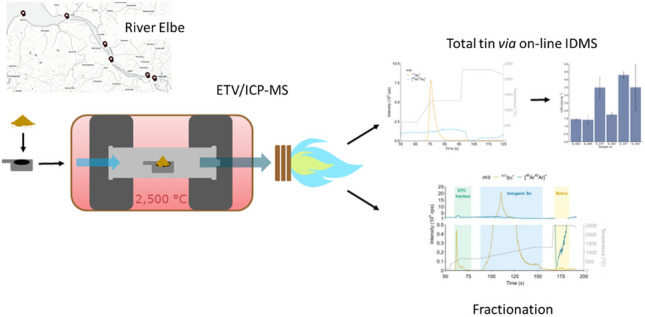

**Supplementary Information:**

The online version contains supplementary material available at 10.1007/s00216-025-06064-y.

## Introduction

Tributyltin (TBT) has been widely used as the main biocidal ingredient in anti-fouling paint on ship hulls since the 1970s [1]. Besides other applications such as wood preservatives, this widespread use as an anti-fouling agent in ship hull paint has led to the release of organotin compounds (OTCs) into the environment. Toxic effects of TBT on aquatic life, including non-target organisms, have been observed at low concentrations of 1–2 ng L^−1^,^1 ^with imposex in gastropods being one of the most frequently described consequences [2, 3]. As a result, the International Maritime Organization (IMO) banned TBT from its application on ship vessels in 2008 [4]. However, the production and sales of TBT-containing vessel paint are ongoing [5], and imposex associated with TBT pollution is still reported in less regulated areas [3, 6]. Despite overall decreasing levels, environmentally relevant TBT concentrations are still found in sediment samples of hotspot areas in the Baltic Sea and Portuguese margin (up to 333.3 ng g^−1^ dry weight for Sn, [7] and 41 ng g^−1^, [8] respectively). Additionally, TBT contamination is correlated with the enrichment of inorganic Sn in sediments [9]. Limited data on the environmental significance of increased tin levels in sediments have been published. However, while inorganic tin is generally considered non-toxic, it may be used to elucidate historical TBT input [9].

The total tin content in sediment samples is commonly determined via inductively coupled plasma-mass spectrometry (ICP-MS) after acid digestion [9]. Solid sampling is advantageous because it enables direct sample introduction into the ICP-MS system. Hence, tedious and time-consuming sample preparation procedures with hazardous chemicals are avoided. Furthermore, the risk of error from sample loss, e.g., in case of volatilization, and contamination is reduced. Speciation analysis of OTCs involves a multi-step sample preparation procedure prior to separation of the individual elemental species by chromatographic separation techniques, most commonly gas chromatography (GC), combined with a sensitive detection method [1, 8, 10, 11]. For solid sampling by electrothermal vaporization (ETV), speciation analysis has been reported for sulfur species in coals [12] and methylmercury and inorganic mercury in biological materials [13]. After loading and transferring a sample boat into the ETV furnace, the individual vaporization of different chemical species is realized through a suitable temperature program.

One major challenge in quantification with solid sampling techniques, such as ETV, is matrix effects originating from the co-volatilization of matrix components and altered analyte transport efficiency (TE) [14]. External calibration is feasible with certified reference materials (CRMs), e.g., for determining Hg in sludge samples [15]. However, CRMs with a suitable matrix and the required certified concentration range of the analyte are not available in every case. Alternative calibration strategies to compensate for these effects involve (i) internal standardization in external approaches with dried aqueous solutions [16, 17], (ii) standard addition [18], or (iii) matrix removal through pre-treatment steps [19]. Isotope dilution mass spectrometry (IDMS) has the advantage of providing accurate, quantitative results while being independent of matrix effects for the most part. The quantification principle is based on measuring the alternated analyte isotope ratio in the sample through the known quantity of an isotopically enriched spike. In combination with ETV, IDMS was first reported by Vanhaecke et al. in 1997 to determine Se or Cd in different solid CRMs, e.g., estuarine sediment [20]. Thereby, an aqueous spike solution was dried in the sample boats prior to the sample loading [20]. In other approaches, the spike was added during sample preparation [21, 22].

For the speciation analysis of OTCs in sediment samples, isotopically enriched forms of the analytes were added in the extraction procedure prior to analysis via GC/ICP-MS [11]. This allowed the monitoring of species transformation during sample preparation. However, performing species-specific IDMS has the disadvantage of relying on the availability of isotopically enriched species that are either labor-intensive to synthesize or expensive when purchased commercially. Species-unspecific IDMS as an on-line approach for speciation analysis (on-line IDMS) was introduced by Rottmann and Heumann in 1994 [23], whereby a species-unspecific spike was continuously added to the elemental species separated by high-pressure liquid chromatography. Since then, on-line IDMS has emerged as an accurate and matrix-independent calibration approach for hyphenated ICP-MS techniques, e.g., capillary electrophoresis/ICP-MS [24], laser ablation/ICP-MS [25], and ETV/ICP-MS [13, 26]. For ETV, the spike addition was realized by vaporizing isotopically enriched mercury in an in-house-built apparatus and directing the vapor through the ETV furnace during measurements. Thus, the methods’ applicability is limited by the vaporability of the spike element and the availability of the required equipment setup.

In our study, we aimed to develop an on-line IDMS calibration method for analyzing solid environmental samples that applies to a wide range of elements and is compatible with commercially available instruments. Method validation was carried out via the total tin content of a sediment CRM, followed by determining the total Sn content of sediment samples along the River Elbe. The sampling campaign encompassed the industrial and urban harbor sites in the City of Hamburg, where high contamination was expected, to the river mouth. Additionally, we investigated the potential of solid sampling ETV for OTC fractionation analysis.

## Experimental

### Chemicals

Tributyltin chloride (95.5%), dibutyltin dichloride (DBT, 95.5%), butyltin trichloride (MBT, 94.5%), and triphenyltin chloride (TPhT, 98.0%) were purchased from Sigma-Aldrich (Steinheim, Germany). Ethanol (99.8%) was purchased from Honeywell (Seelze, Germany). Water was purified by a Milli-Q water purification system (Merck Millipore, France). Hydrochloric acid (HCl, 30%) from Th. Geyer (Renningen, Germany) was sub-boiled prior to use. Gold nanoparticles were purchased as stabilized suspensions in citrate buffer from Sigma-Aldrich (80 nm diameter, Steinheim, Germany) and Merck KGaA (60 nm diameter, Darmstadt, Germany).

^117^Sn-enriched elemental tin (92.0 ± 0.4%) from Techsnabexport (Moscow, Russia) was dissolved and diluted to 18.4043 µg g^−1^ in 20% HCl. The concentration of the isotopically enriched stock solution and the isotopic composition were determined prior to use.

### Certified reference materials and real-world samples

**Fig. 1:**
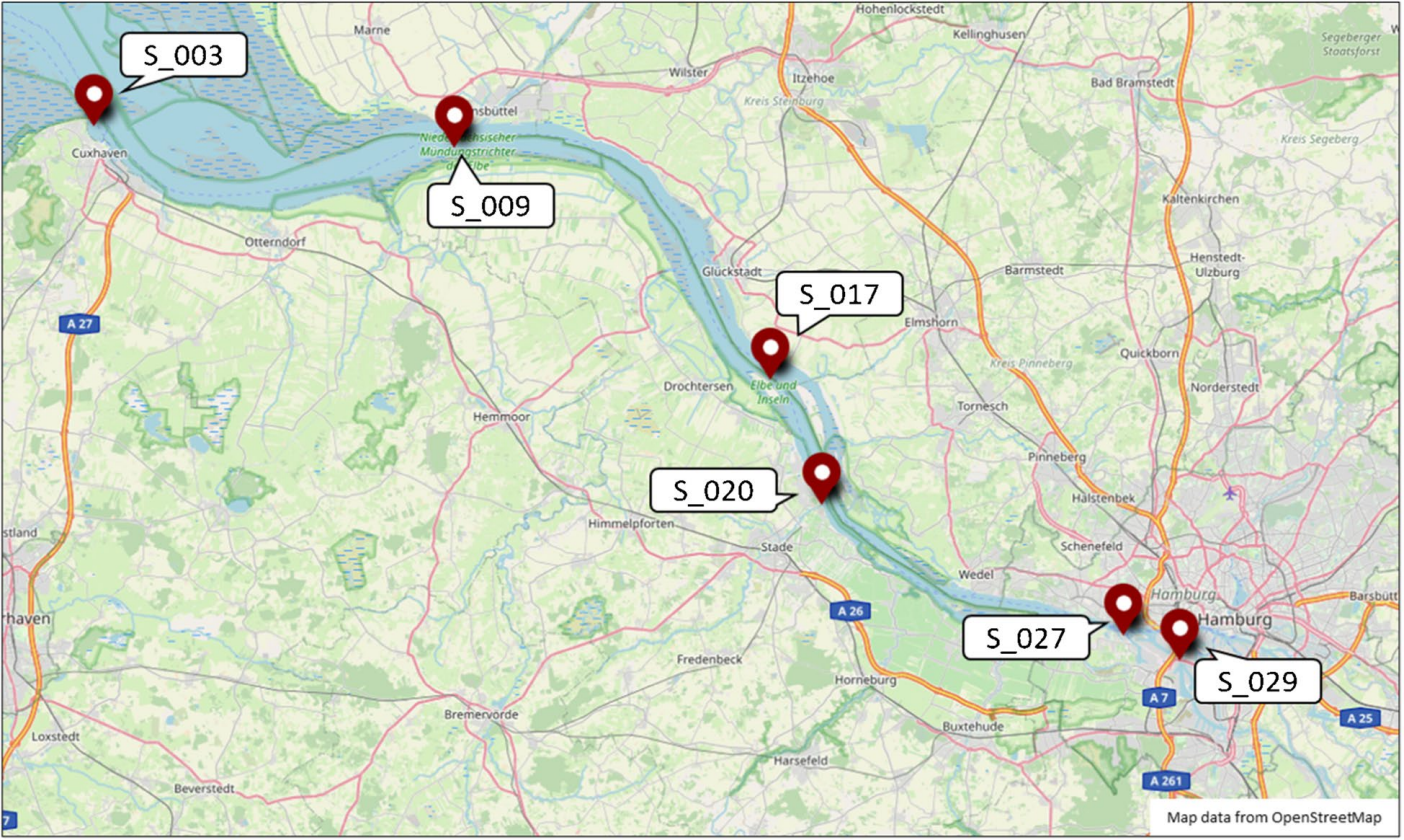
Map of the sediment sampling locations. The map was created using OpenStreetMap (openstreetmap.org/copyright)

The CRMs BCR-646 and BCR-277R from the Institute for Reference Materials and Measurements (Geel, Belgium) were used for method development and validation. Real-world sediment samples from the tidal Elbe were collected during a ship sampling campaign in August 2015 conducted by the Helmholtz Center hereon [27]. Samples from the mouth of the river (S_003, river km 727) to Hamburg (S_029, river km 624) were selected for quantification via on-line IDMS (Fig. 1). A sample list with Global Positioning System (GPS) coordinates is provided in the Supplemental Information (SI, Table S1). The sediment top layer (5 to 10 cm) was obtained using a custom-made box corer, and three or more individual samplings were subsequently homogenized and deep-frozen [27]. The homogenized sediment samples were freeze-dried (Christ Gefriertrocknungsanlagen, Osterode, Germany) [27] and sieved over a cascade of polyamide sieves (Atechnik, Leinburg, Germany) to obtain the < 2 mm grain size fraction. To ensure the homogeneity of the sample aliquots introduced into the ETV furnace, sediment samples were milled using a mixer mill MM 400 from Retsch GmbH (Haan, Germany) equipped with a frequency of 30 s^−1^ for 20–90 s. The milling time was chosen depending on the sediment texture until an optically homogeneous sample was obtained.

### Instrumentation

**Fig. 2:**
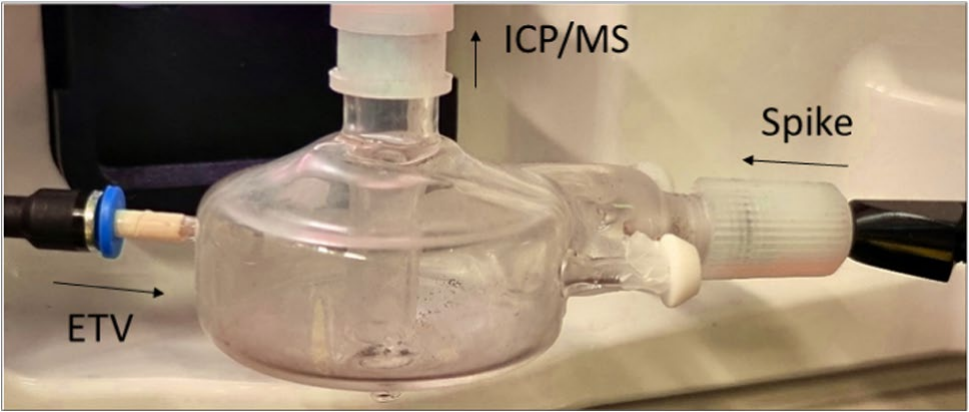
Photo of the modified cyclon spray chamber used for merging of the spike and ETV sample aerosol

A commercially available ETV system ETV-4000d with an autosampler AD30 (Spectral Systems, Fürstenfeldbruck, Germany) was coupled to a quadrupole-based ICP-MS Thermo Scientific iCAP Qc (Thermo Fisher Scientific, Bremen, Germany). The spike was introduced via a modified cyclonic quartz spray chamber from Thermo Fisher Scientific (Bremen, Germany) equipped with an X175 nebulizer from Burgener Research Inc. (Mississauga, Ontario, Canada) as shown in Fig. 2. To accomplish a high TE, the waste port of the spray chamber was sealed, and a port for the sample gas introduction from ETV was added. The constant proliferation of the spike solution was ensured using a 1 mL Hamilton glass syringe (Hamilton Company, Gräfling, Germany) and a syringe pump from Landgraf Laborsysteme HLL GmbH (Langenhagen, Germany).

The ICP-MS was equipped with an alumina sampler and nickel skimmer cone for high matrix (Glass Expansion, Weilburg, Germany), as well as a quartz torch and injector (2.5 mm inner diameter) from Thermo Fisher Scientific (Bremen, Germany). For IDMS measurements, the sampling depth was tuned to 8 mm, and the gas pressure at the low-flow nebulizer was optimized to 65 psi (4.5 bar) for an argon flow of 1.04 L min^−1^. Detailed operating conditions of the setup are given in Table 1. The TE of the spike introduction system was determined daily before IDMS measurements via the particle size method using gold nanoparticles [28]. The internal pyrometer of the ETV system was calibrated daily with an external pyrometer PKL 38 AF 1 (KELLER HCW GmbH, Ibbenbüren, Germany) prior to measurements. As recommended by the manufacturer, carrier and bypass gas flow rates of 0.142 L min^−1^, and 0.420 L min^−1^, respectively, were applied at the ETV furnace. Trifluoro methane (GHC, Hamburg, Germany) was added as reaction gas with an optimized flow rate of 0.5 mL min^−1^ to the carrier gas flow to prevent the formation of tin carbides. ^117^Sn and ^122^Sn were acquired for IDMS analysis, whereas ^121^Sb and ^125^Te were monitored to observe possible spectral interferences on ^122^Sn, and [^40^Ar]_2_^+^ to detect non-spectral interferences. [17] For the sensitive detection of an OTC fraction, ^120^Sn was measured.

**Table 1:** Operating conditions of the ETV/ICP-MS system for IDMS measurements

Parameter	Value
ICP-MS Instrument	iCAP Qc
Cones	Al sampler cone, Ni skimmer cone for high matrix
Plasma power (W)	1500
ICP gas flows (L min^−1^)	0.65 (auxiliary gas), 14 (cool gas)
Sampling depth (mm)	8.0
ETV system	ETV-4000d equipped with autosampler AD30
Sample boats and tubes	Pyrolytically coated graphite, size “maxi”
ETV gas flows (L min^−1^)	0.142 (carrier gas), 0.420 (bypass gas)
Isotopes monitored	^117^Sn, ^120^Sn, ^122^Sn, ^121^Sb, ^125^Te, [^40^Ar]_2_^+^ with 30 ms dwell time each

### Procedures

Approximately 1 mg of the sediment samples (CRMs and real-world samples) were weighed into pyrolytically coated graphite boats obtained from Spectral Systems (Fürstenfeldbruck, Germany) and transferred into the ETV furnace via the autosampler. ETV temperature programs for determining the total tin content and OTC fractionation analysis, respectively, are given in Table 2. A UMT5 microbalance (*d* = 0.0001 mg, min. 0.01 mg) from Mettler-Toledo GmbH (Gießen, Germany) was used for on-line IDMS. Fractionation analysis was performed with a Sartorius MC1 Research RC 210 D analytical balance (Sartorius AG, Göttingen, Germany). For spiking experiments, aliquots of the ethanolic OTC solutions were pipetted directly onto the sediment material and dried under ambient conditions prior to measurement.

**Table 2:** Optimized temperature programs

Step	Determination of the total tin content (P1)	OTC fractionation analysis (P2)
Conditioning	5 s at 20°C^a^, 20°C^a^ to 80 °C in 20 s, 30 s hold time^b^	
Vaporization	600 °C to 1300 °C in 10 s, 20 s hold time	350 °C to 650 °C in 10 s, 20 s hold time,
		650 °C to 1300 °C in 60 s, 20 s hold time
Cleaning	1300 °C to 2300 °C in 1 s, 20 s hold time	1300 °C to 2500 °C in 1 s, 20 s hold time

^a^Ambient condition (no power applied)

^b^In 1 s to 600 °C (P1) and 350 °C (P2), respectively. An additional 10 s hold time of 600 °C for ETV program P2

Conditions for on-line IDMS were chosen to establish a fast-screening method. For this purpose, the spike flow was kept constant throughout the measurements. Mass discrimination factors *K* were determined by analyzing the CRM BCR-277R in triplicate on each measurement day without spike introduction.

### Data processing

Raw data CSV files were exported from the ICP-MS software Qtegra (Thermo Fisher Scientific, Bremen, Germany) and processed using an in-house-built *R*script [29]. The data treatment encompassed (i) calculation of the isotope ratios, (ii) mass bias correction, (iii) calculation of the mass flow, and (iv) peak integration. The sample mass flow ${\dot{M}}_{s}(t)$ in pg s^−1^ was obtained as a function of time after Eq. 1 adapted from Rottmann and Heumann.^23^

1 $${\dot{M}}_{s}(t) ={\dot{M}}_{sp}\bullet \frac{{h}_{sp}^{117}-{R}_{corr}(t)\bullet {h}_{sp}^{122}}{{h}_{s}^{122}\bullet {R}_{corr}\left(t\right)-{h}_{s}^{117}}$$

Isotope ratios *R*(t) of ^117^Sn and ^122^Sn were formed point-by-point and corrected for the mass bias with *K*. ${\dot{M}}_{sp}$ is the mass flow of the spike in pg s^−1^ calculated from the mass concentration of ^117^Sn in the spike solution, the spike flow rate, and the TE. A modified RIKILT single particle calculation tool (version 2) was used to evaluate single particle signals [30]. Detailed equations are found in the SI (Eq. S1–S3). ${h}_{sp}^{117}$ and ${h}_{sp}^{122}$ are the abundances of the isotopes ^117^Sn and ^122^Sn in the spike solution, respectively. For the samples, a natural isotopic composition (${h}_{s}^{117}, {h}_{s}^{122}$) was presumed. The contribution of the isotope ratio of the blank was considered negligible compared to the expected relative standard deviation caused by the small sample amount [20]. Hence, no blank correction was performed. Peak integration followed by baseline correction provided the mass of tin *m*_s_(pg) in the sample. The limit of detection (LOD) and quantification (LOQ) were determined as three and ten times the standard deviation from ten consecutive spiked blank measurements, respectively [13, 21].

## Results and discussion

### Method development and validation

#### On-line IDMS

**Fig. 3:**
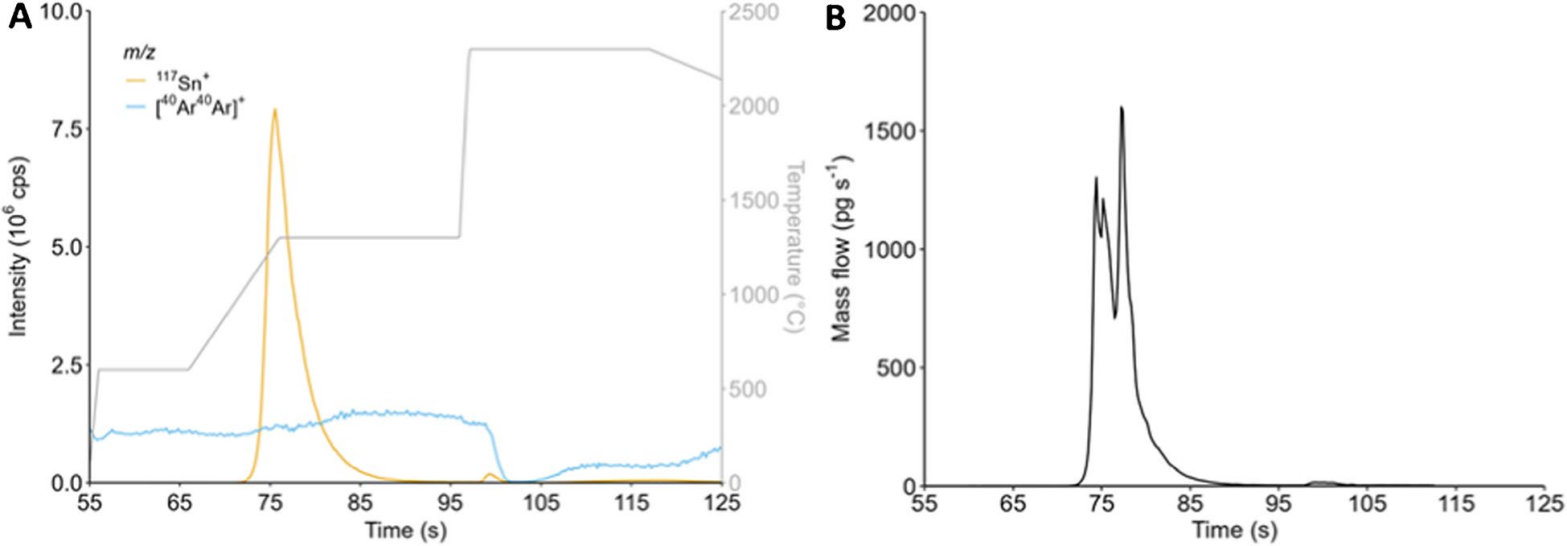
**A** ETV/ICP-MS time scan of BCR-277R with a reaction gas flow rate of 0.5 mL min^−1^ applying the developed ETV temperature program for determining the total tin content (P1). The temperature program is shown in gray.** B** Mass flow diagram of an ETV/ICP-MS measurement of BCR-277R (*m* = 1.0119 mg)

The CRM BCR-277R was selected to develop and validate a method for quantifying total tin in sediment samples via ETV/ICP-MS. An example time scan applying the temperature program for the total tin determination is depicted in Fig. 3A. The absence of spectral interferences on ^122^Sn^+^ was confirmed using ^121^Sb^+^ and ^125^Te^+^. Non-spectral interferences were monitored via the [^40^Ar^40^Ar]^+^signal [20], which remained relatively constant during the tin vaporization starting at ~ 70 s, equaling a temperature of 880 °C. However, fluctuation between different runs, e.g., due to matrix deposits on the cones, was observed. Calibration with external liquid standards was not feasible, presumably caused by co-vaporization of matrix components [26]. To correct for these effects, an on-line IDMS method was developed. Evaluation of the on-line IDMS data, including calculation of the isotope ratios, mass bias correction, and mass flow determination, followed by peak integration, was adapted from the literature [23]. An example calculation is provided in the SI. The separation of analyte and major (inorganic) matrix components indicated by a drastic argon dimer signal dip was achieved by the optimized temperature program P2. Low tin signals during the cleaning step could be caused by residual tin embedded in the silicate matrix, but were considered negligible compared to the inorganic tin peak.

On-line IDMS is known as an accurate quantification method [23]. However, elevated relative standard deviations (RSDs) were expected due to sample inhomogeneity and the low sample amount (~ 1 mg) introduced via ETV. A total tin mass fraction of 6.5 ± 1.8 mg kg^−1^ (informative value) is specified for BCR-277R. Results obtained via our on-line IDMS approach yielded a tin mass fraction of 6.6 mg kg^−1^, equaling a recovery of 106% (*n* = 3), and remained within the given uncertainty. An RSD of 14% was assessed as acceptable for a fast-screening method. A comparable RSD of 18.3% (*n* = 6) is reported for quantifying mercury in river sediment by on-line ETV/ICP-IDMS with a sample weight of ~ 1 mg.^26^ The LOD and LOQ were estimated for a theoretical sample mass of 1 mg to 0.002 mg Sn kg^−1^ and 0.008 mg Sn kg^−1^, respectively. Lower limits could be accomplished by acquiring Sn isotopes of higher abundance, e.g., ^120^Sn (*h* = 32.59%) and ^118^Sn (*h* = 24.23%). However, adequate figures of merit for quantifying total tin in sediment could be achieved by using ^117^Sn and ^122^Sn with isotopic abundances of 7.68% and 4.63%, respectively. A similar LOD of 0.006 mg Hg kg^−1^ is reported in the literature^13, 26^ for determining inorganic mercury via the ^202^Hg isotope (*h* = 29.86%) with a ^200^Hg-enriched spike (*h* = 29.86%).

#### Fractionation analysis

The potential of solid sampling ETV for the speciation analysis of metalorganic compounds has been shown by Gelaude et al. for mercury species [13]. To evaluate the separation capability of ETV/ICP-MS for tinorganic species, the CRM BCR-646 was selected. The sediment material contains certified mass fractions of TBT and the dealkylation intermediates DBT and MBT, as well as TPhT and its degradation products diphenyltin (DPhT) and phenyltin (MPhT). No information was provided on inorganic tin content by the manufacturer. However, inorganic tin of geological origin and anthropogenic sources was expected at levels above the LOD. Two tin peaks were achieved through a temperature ramp with a gradual temperature increase of 10 °C s^−1^ (Figure S1 in the SI). Hereby, the acquisition of the ^117^Sn isotope with an abundance of 7.68% provided sufficient signal intensities, while no relevant spectral interferences were anticipated.

**Fig. 4:**
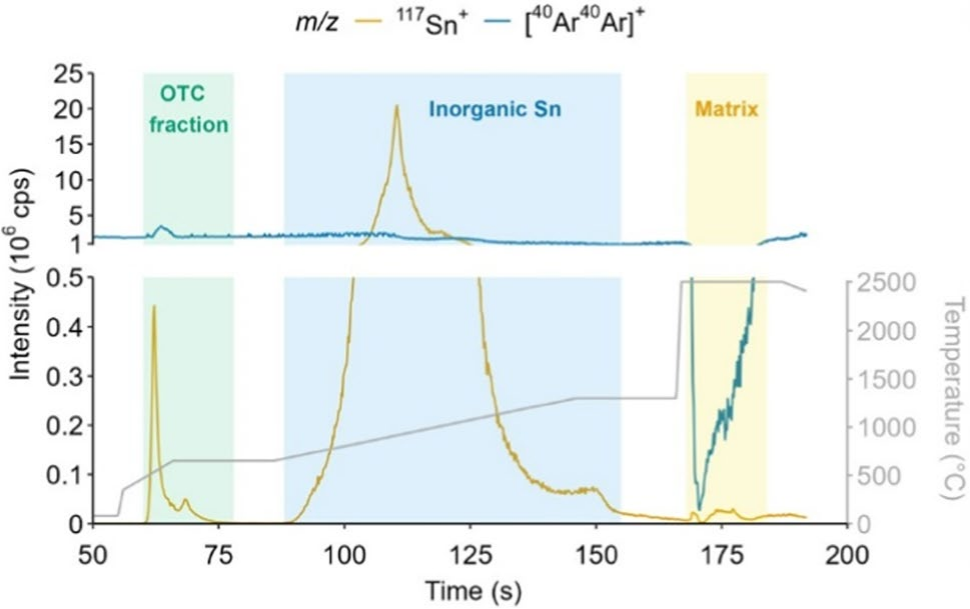
ETV/ICP-MS time scan of BCR-646 with a reaction gas flow rate of 0.5 mL min^−1^ applying the developed multi-step temperature program (P2). The temperature program is shown in gray

To obtain baseline-separated peaks, a multi-step temperature program was developed (Fig. 4). The constant analyte release from the matrix without establishing an equilibrium was accomplished via temperature ramps. The peak detected during the first vaporization step was assigned to an organotin fraction. The hereby applied temperature range (350 to 650 °C) was higher than the boiling points of the declared OTCs, ranging from 93 °C at 13 hPa (MBT trichloride) to 333–337 °C (decomposition of DPhT dichloride). ^117^Sn peaks were not observed at lower temperatures, presumably caused by the actual structure in the sediment sample and its interaction with the matrix. In sediments, OTCs are primarily present as hydrophobic hydroxyl complexes and cations associated with organic matter and clay [1, 2]. These findings follow observations for pyrolysis-GC measurements, where pyrolysis temperatures > 300 °C were required to detect organic contaminants in real-world sediment samples [31]. The transfer time from the ETV furnace to the detector must be considered when discussing the temperature program. A second tin peak was attributed to inorganic tin due to its appearance at higher temperatures (650 to 1300 °C) and intensity (~ 40 times higher than the OTC fraction peak). A small peak shoulder could be caused by tin embedded in the inorganic matrix. Both tin peaks were accompanied by variations in the argon dimer signal. The small [^40^Ar^40^Ar]^+^ peak at ~ 65 s could be explained by co-vaporization of other possibly ^80^Se-containing organic matrix components. The observed signal decrease during the inorganic tin detection is presumably caused by the release of inorganic matrix components, leading to plasma cooling [17]. Similar observations are reported by Gelaude et al. [13] during the vaporization of methyl and inorganic mercury from a lobster sample.

**Fig. 5:**
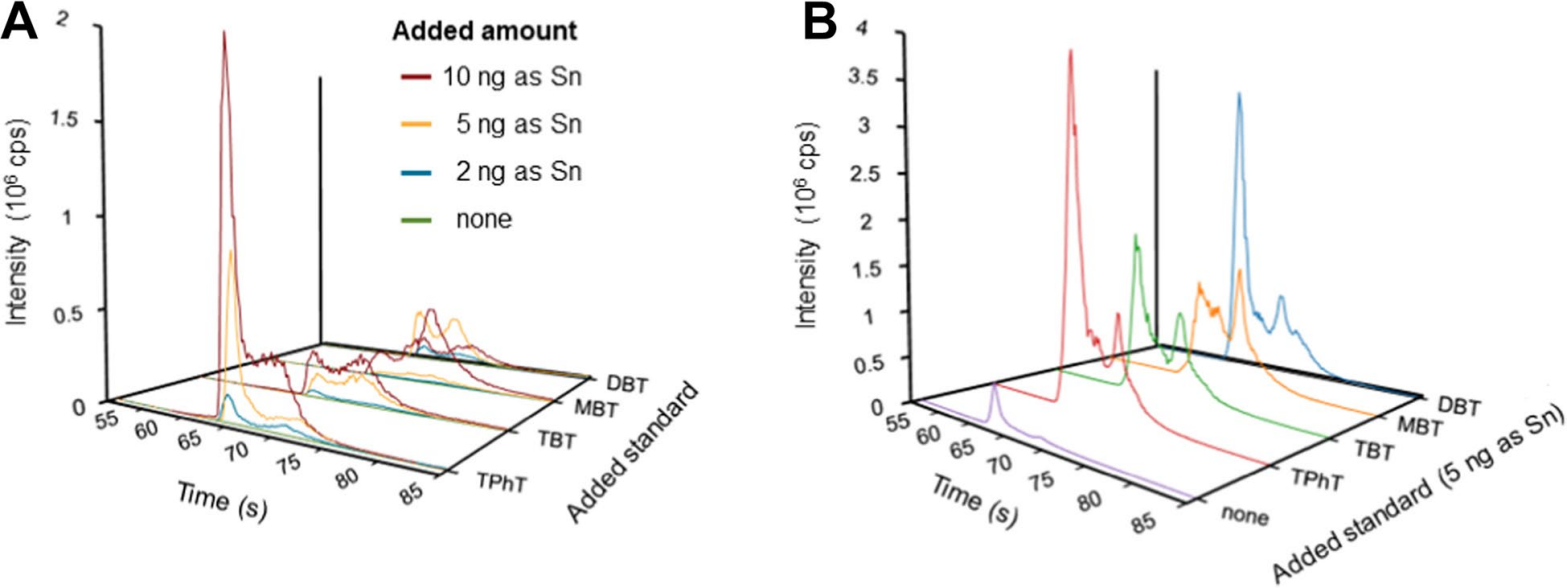
Excerpts of the ETV/ICP-MS time scans of **A** BCR-277R without added OTC standard and spiked with 2, 5, and 10 ng (as Sn) of TPhT, TBT, MBT, and DBT, respectively, and **B** BCR-646 without added OTC standard and 5 ng (as Sn) of each OTC, applying temperature program P2

BCR-277R, without any information on OTCs in the certificate, was used to validate the identity of the OTC fraction peak by spiking experiments with ethanolic OTC solutions of 2–10 ng (as Sn) of each compound. Without any added standard, no peak was apparent in the “OTC fraction” window (56–86 s), indicating the absence of detectable quantities of OTC in the sample (Fig. 5A).

The proposed peak assignment to an OTC fraction was confirmed by an approximate correlation of the peak signal intensity between 56 and 86 s with the added amount of each OTC. Thereby, retention of the vaporization by contact with the graphite boat could be the cause, as observed for the direct measurement of OTC solutions. Respective peak shapes depend on the individual OTC; e.g., for TPhT, a peak with a smaller shoulder was found, while for TBT and DBT, two equal peaks were detected. Hence, peak splitting within the “OTC fraction” window could not be assigned to individual OTCs or OTC groups such as trisubstituted or butyltins. However, different association forms to matrix components, e.g., van der Waals forces and ionic interactions to organic matter and clay, respectively, could affect the peak shape [1, 2]. Hence, the vaporization behavior of OTCs in the ETV furnace seems to be largely influenced by matrix effects.

When spiked with different OTCs, no additional peak in the ETV/ICP-MS time scans and signal enhancement in the “OTC fraction” time window was observed for BCR-646 (see Fig. 5B). Hereby, a comparable peak shape was detected for the individual OTCs, which could arise due to similar matrix interactions as discussed above. These findings confirm the assignment of OTCs to the first peak. However, quantifying the OTC fraction peak with the developed on-line IDMS method yielded a recovery of 3.2% for BCR-646. Consequently, the first peak represents only a small fraction of the actual OTC content in the CRM. This could result from matrix effects and interactions with the ETV graphite tube hindering the separation of OTCs from inorganic tin.

### Investigation of sediment samples from the River Elbe

#### Total tin content

**Fig. 6:**
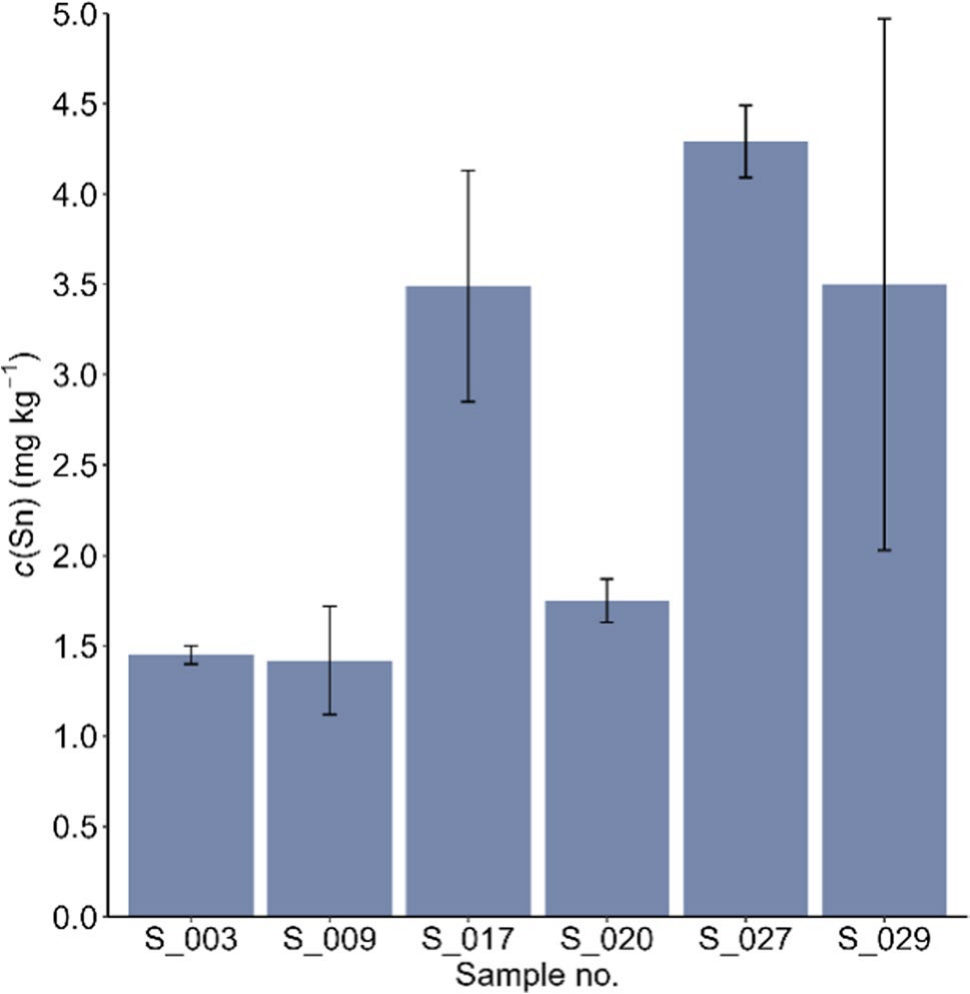
Total tin mass fractions of River Elbe sediment samples analyzed via on-line IDMS ETV/ICP-MS

River Elbe sediment samples were analyzed by ETV/ICP-MS using the developed on-line IDMS calibration approach. Total tin mass fractions varied from 1.42 to 4.29 mg kg^−1^ along the River Elbe course (Fig. 6). The lowest tin content was quantified near the mouth of the river at km 727 (1.42 mg kg^−1^, S_003) and 698 (1.42 mg kg^−1^, S_009). Elevated contents (4.29 mg kg^−1^, S_027 and 3.50 mg kg^−1^, S_029) for sediment from sampling locations at the city of Hamburg (Seemannhoeft; river km 629, and Koehlbrand; river km 624) can be correlated with industrial activities and historical tin introduction via the River Mulde.^32^ Generally, an increase in the total tin mass fraction from the North Sea to the City of Hamburg could be observed, except for sample S_017 (Tonne 91, gruen; river km 665). With a mass fraction of 3.49 mg kg^−1^, a 50% higher value than for the 10 km distant sampling location Schwinge (S_020, 1.75 mg kg^−1^) was determined. The results follow trends reported in the literature for metal mass fractions and are explainable by the upstream dilution of anthropogenically polluted river sediment with less contaminated marine sediment via tidal currents [27, 33, 34].

Multi-elemental and isotopic data are available in the literature for real-world sediment samples investigated in this work [27, 33]. Reported results can be affected by differences in the analyzed grain size fractions (< 63 μm grain size fraction [27, 33], this work: < 2 mm). However, metal mass fractions and isotope signatures provide valuable information about the contamination load of the sediment samples. A local peak in the metal mass fractions of Cd, Pb, Cu, and Zn (> 1.8 times higher than the surrounding area) is reported for sample S_017 in the literature [27, 33]. Elevated metal concentrations are reported for the respective sampling area “Schwarztonnensand” [35]. A reduced flow in this region leads to the sedimentation of suspended particulate matter, which is not retained in the harbor area of Hamburg during high freshwater flow [35]. Historical data from sampling campaigns in 1992–1998 reveal an overall reduction of the tin content between river km 730 and 650 by more than three times, presumably because of reduced intakes and subsequent dilution over time [36]. Similar observations are reported by Zimmermann et al. for zinc [33].

It must be noted that RSDs (*n* = 3) ranged from 3.4% (S_003) to 42.2% (S_029). Elevated RSDs were presumably caused by sample inhomogeneity and the small sample amount (~ 1 mg) introduced via ETV. Lower RSDs could be achieved by further optimization of the milling process and a higher number of repetition measurements, respectively. In this work, a visual check for homogeneity was considered sufficient for a case study. However, both measures would elongate the overall measurement time.

#### OTCs fractionation

**Fig. 7:**
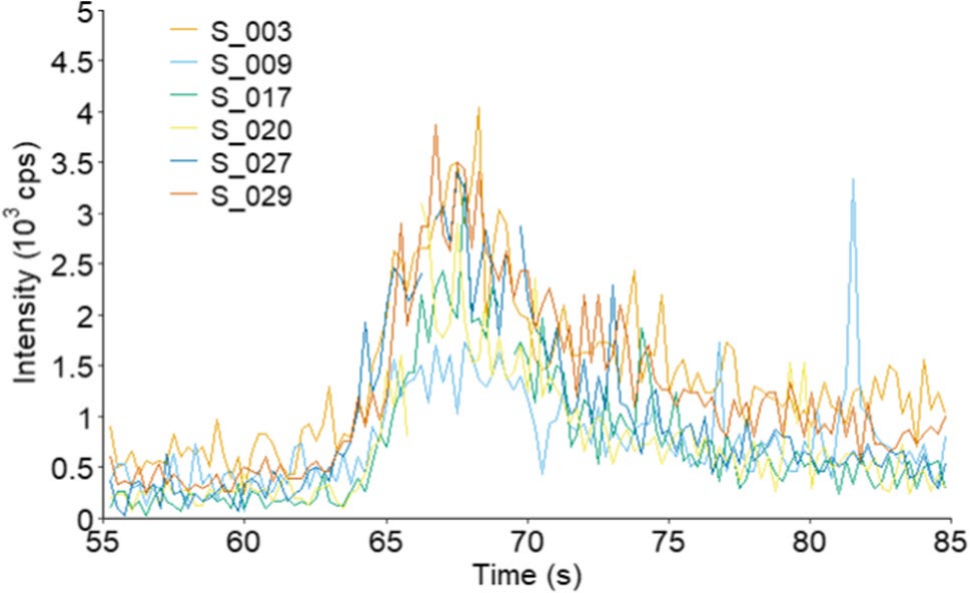
Excerpts of the ETV/ICP-MS time scans in the River Elbe samples’ OTCs-fraction time window (56–86 s, 350–650 °C)

To investigate the detectability of an OTC fraction along the tidal Elbe, real-world samples were qualitatively scanned with the multi-step temperature program developed for fractionation experiments (P2). The ^120^Sn isotope was monitored to attain sensitive detection. A peak in the OTC fraction window (56–86 s, 350–650 °C) was observed for all sediment samples (Fig. 7), indicating the presence of OTCs in the surface sediments. Assuming similar recoveries were obtained for the sample sediments as for the CRM BCR-646, sensitivity could be further improved by increasing the OTC fraction yield. However, we were able to show the fractionation method’s applicability to real-world OTC levels.

For tidal Elbe sediment from sampling campaigns from October 1992, 1995, and 2006, published OTC levels in the µg g^−1^ range decrease towards the river mouth.^32, 34^ As described for tin, the polluted material is presumably diluted by the upstream transport of less contaminated North Sea sediment. However, enhanced concentrations are still reported in the estuary at Cuxhaven.^32^An increase in OTC mass fractions from the 1990 s to 2006 is likely caused by ongoing use and subsequent accumulation in the sediment [32, 34]. Reduced OTC contents are expected for the samples in this work due to legislative restrictions. However, an OTC fraction was still detectable for a sample in the estuary at Cuxhaven (S_003), where a strong dilution with marine sediment is anticipated.

By this, on-line IDMS ETV/ICP-MS could be applied as a complementary approach to species-specific GC/MS methods, which are necessary to obtain detailed toxicological information. Similar to determining the total chromium content to reduce the sample amount for the analysis of toxic Cr(VI), a fast screening for OTCs prior to more time-consuming and laborious species-specific analysis could reduce the overall analysis time and chemical consumption.

## Conclusion

In this work, we successfully combined ETV/ICP-MS with on-line IDMS for quantifying metal-based pollutants in environmental samples. Continuous spike introduction was realized using a modified cyclonic spray chamber and a low-flow nebulizer. Conditions were chosen to implement a fast-screening method yielding a recovery of 106% (*n* = 3) for determining the total tin mass fraction in a sediment reference material with an RSD (14%) comparable to literature values. Quantification via the ^122^Sn isotope using a ^117^Sn-enriched spike resulted in a LOD of 0.002 mg Sn kg^−1^ and a LOQ of 0.008 mg Sn kg^−1^. By applying the method to River Elbe sediment samples from the river mouth (river km 727) to the City of Hamburg (river km 623), total tin mass fractions from 1.42 to 4.29 mg Sn kg^−1^ were obtained. A general increase in the tin content from the estuary along the river course agreed with reported historical data and metal mass fractions. Enhanced RSDs (3.4–42.2%) were ascribed to sample inhomogeneity and the small sample mass, respectively. Further improvement could be achieved by optimizing the milling process prior to solid sampling.

We further showed that separating organotin pollutants from inorganic tin in sediment via an ETV program is partially possible. A multi-step temperature program was developed using a CRM sediment containing six OTCs. The method was validated by spiking sediment reference materials with different OTC standards. Quantifying the peak assigned to the OTC fraction yielded only a small portion of the certified mass fraction (3%). However, an OTC fraction was detectable in all tidal Elbe sediment samples. To establish ETV/ICP-MS as a screening method complementary to species-specific analysis, further improvement of the fractionation yield is required. Additionally, the applicability to other matrices could be tested since a strong matrix interaction could explain the observed analyte retention. 

## Supplementary Information

Below is the link to the electronic supplementary material.

Supplementary file1 (DOCX 168 KB)

## Data Availability

All data will be made available upon request to the corresponding author.
