# Supplementary material for: Tin fractionation analysis in sediment samples via on-line ID ETV/ICP-MS
Source: Anal Bioanal Chem. 2025 Aug 21;418(16):5097–106. doi: 10.1007/s00216-025-06064-y (PMC13424077; doi:10.1007/s00216-025-06064-y)
Supplement: Supplementary file 1 — (DOCX 168 KB) [file 216_2025_6064_MOESM1_ESM.docx]

Supplementary Information

Tin Fractionation Analysis in Sediment samples via on-line ID ETV/ICP-MS

Vera M. Scharek^1^, Jens Pfeifer^1^, Jochen Vogl^1^, Heike Traub^1^, Björn Meermann^1*^

^1^ Federal Institute for Materials Research and Testing (BAM), Division 1.1 – Inorganic Trace Analysis (ITALab), Richard-Willstätter-Straße 11, 12489 Berlin

*Corresponding author: PD Dr. habil. Björn Meermann, Email: bjoern.meermann@bam.de

Keywords: Tin, Organotin compounds, Sediment, Fractionation, On-line IDMS, ETV/ICP-MS

Table S1. Sampling information of real-world sediment samples from the River Elbe, including sampling location and date, coordinates, and river kilometer.

| Sample identification | Location name | Sampling date | GPS coordinates | Latitude | Longitude | River kilometer |
| --- | --- | --- | --- | --- | --- | --- |
| S_003 | Kugelbake | 08/03/2015 | N 53°53'31" E 08°41'53" | 53.8885 | 8.6922 | 727.0 |
| S_009 | Tonne 57 | 08/04/2015 | N 53°52'40" E 09°06'30" | 53.8733 | 9.1050 | 698.0 |
| S_017 | Tonne 91 gruen | 08/05/2015 | N 53°42'93" E 09°28'05" | 53.7155 | 9.4675 | 665.0 |
| S_020 | Schwinge | 08/05/2015 | N 53°37'78" E 09°31'62" | 53.6297 | 9.5270 | 655.0 |
| S_027 | Seemannshoeft | 08/05/2015 | N 53°32'42" E 09°52'38" | 53.5403 | 9.8730 | 628.8 |
| S_029 | Koehlbrand | 08/05/2015 | N 53°31'40" E 09°56'23" | 53.5233 | 9.9372 | 622.6 |

The following equations were used to obtain the variables in the sample mass flow $\dot{M}_{s}(t)$ equation.

Three aliquots of the CRM BCR-277R without spike introduction were measured to determine the mass discrimination factor *K* as follows.

$K=\frac{R_{true}}{R_{m}}=\frac{h_{s}^{117}\cdot h_{m}^{122}}{h_{s}^{122}\cdot h_{m}^{117}}$ (S1)

Hereby, the isotope ratio ^117^Sn/^122^Sn *R*_true_ is calculated from the natural tin isotope abundances ($h_{s}^{117}$, $h_{s}^{117}$) and the measured ratio *R*_m_ is the quotient of the integrated peak areas of ^117^Sn ($h_{m}^{117})$ and ^122^Sn ($h_{m}^{122})$, respectively.

Example calculation:

$$K=\frac{0.0768\cdot18156078.01 counts}{0.0463\cdot26600461.76 counts}=\frac{1.659}{1.465}=1.132$$

Corrected isotope ratios *R*_corr_(t) were obtained by multiplying isotope ratios *R*(t) and *K* point-by-point (Eq. S2).

$R_{corr}\left( t \right)=R(t)\cdot K$ (S2)

Example calculation:

$$R_{corr}(80.1 s)=\frac{2019434.4 cps}{745162.698 cps}\cdot1.134=3.073$$

Example figures:


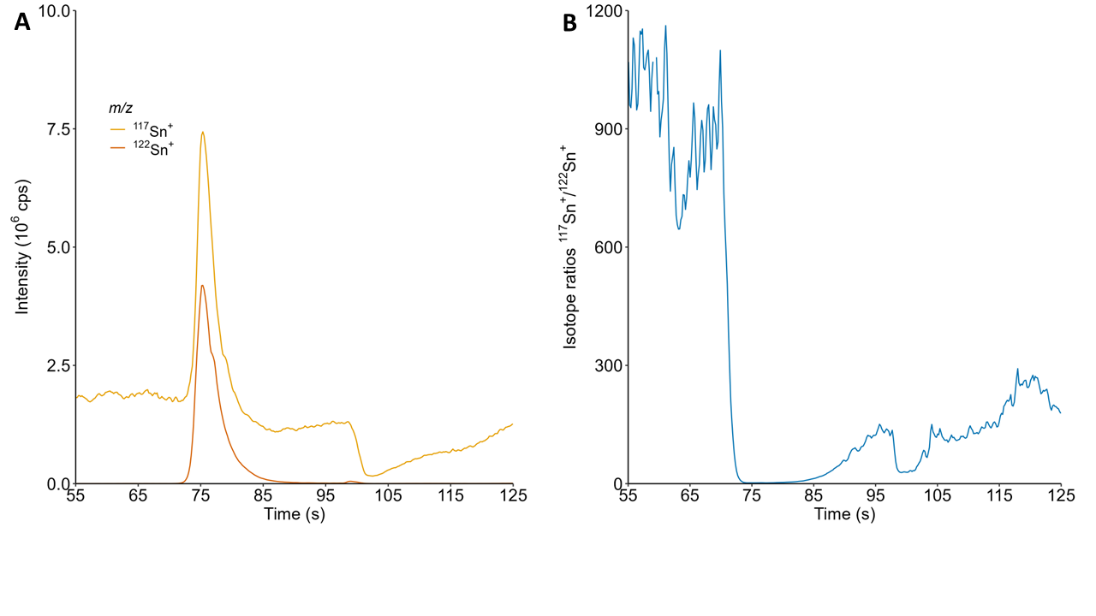


Fig. S1: **A:** Isotope signal profiles of an ETV/ICP-MS measurement of BCR-277R (*m* = 1.0119 mg) with spike (*ρ*(^117^Sn) = 979.09 µg L^-1^) and **B:** the corresponding, corrected ^117^Sn^+^/^122^Sn^+^ isotope ratio profile.

The spike mass flow $\dot{M}_{sp}$ (pg s^-1^) was calculated from the mass concentration of ^117^Sn in the spike solution *ρ*(^117^Sn) (µg L^-1^), the spike flow rate $\dot{V}$ (µL min^-1^) and the transport efficiency $\eta_{trans}$ after Eq. S3.

$\dot{M}_{sp}=\rho({}^{117}{Sn)}\cdot\dot{V}\cdot\eta_{trans}\cdot\frac{1}{60}$ (S3)

Example calculation:

$$\dot{M}_{sp}=979.09 \mu g L^{-1}\cdot7.5 \mu L {min}^{-1}\cdot0.170\cdot\frac{1}{60}=20.8 pg s^{-1}$$

The mass flow was then computed as a function of time after the mass flow equation (Eq. 1).

Example calculation:

$$\dot{M}_{s}\left( 80.1 s \right)=20.8 pg s^{-1}\cdot\frac{91.06-3.073\cdot0.08}{4.63\cdot3.073-7.68}= 288.9 pg s^{-1}$$

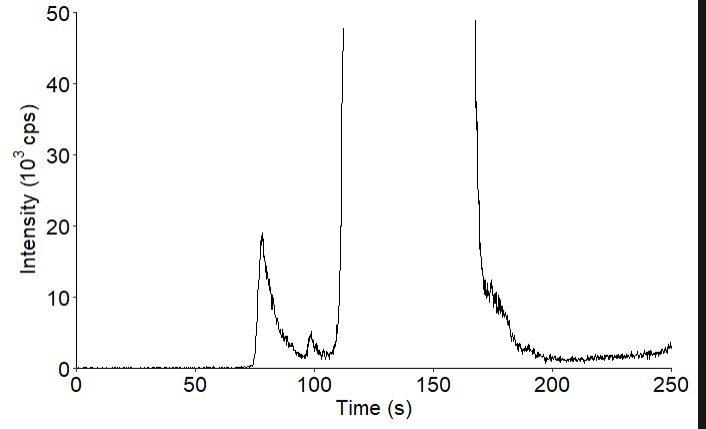


Fig. S2: ^117^Sn ETV/ICP-MS time scan of BCR-646 (~1 mg) measured with a temperature ramp of 10 °C s^-1^ (100 🡪 2500 °C).
